# Supplementary material for: HIVprotI: an integrated web based platform for prediction and design of HIV proteins inhibitors
Source: J Cheminform. 2018 Mar 9;10:12. doi: 10.1186/s13321-018-0266-y (PMC5845081; doi:10.1186/s13321-018-0266-y)
Supplement: Supplementary file 2 — Additional file 2. Source code of HIVProtI web server. [file 13321_2018_266_MOESM2_ESM.zip › HIVprotI_Source-code/hivprotI/weka-3-6-6/doc/weka/core/Matchable.html]

Matchable


---


|  |  |  |
| --- | --- | --- |
| **Overview** \| **Package** \| **Class** \| **Tree** \| **Deprecated** \| **Index** \| **Help** \| **Weka's home** | |  |
| **PREV CLASS**   **NEXT CLASS** | **FRAMES**    **NO FRAMES**     **All Classes** |  |
| SUMMARY: NESTED \| FIELD \| CONSTR \| METHOD | DETAIL: FIELD \| CONSTR \| METHOD |  |


---


## weka.core Interface Matchable

**All Known Implementing Classes:**: J48, J48graft

---

``` public interface Matchable ```

Interface to something that can be matched with tree matching
algorithms.

**Version:**
:   $Revision: 1.6 $

**Author:**
:   Len Trigg (trigg@cs.waikato.ac.nz)

---

| **Method Summary** | |
| --- | --- |
| `java.lang.String` | `prefix()`             Returns a string that describes a tree representing the object in prefix order. |

**Method Detail**

### prefix

```
java.lang.String prefix()
                        throws java.lang.Exception
```

:   Returns a string that describes a tree representing
    the object in prefix order.

    :   **Returns:**: the tree described as a string **Throws:**: `java.lang.Exception` - if the tree can't be computed


---


|  |  |  |
| --- | --- | --- |
| **Overview** \| **Package** \| **Class** \| **Tree** \| **Deprecated** \| **Index** \| **Help** \| **Weka's home** | |  |
| **PREV CLASS**   **NEXT CLASS** | **FRAMES**    **NO FRAMES**     **All Classes** |  |
| SUMMARY: NESTED \| FIELD \| CONSTR \| METHOD | DETAIL: FIELD \| CONSTR \| METHOD |  |


---
